# Transdifferentiation of bovine epithelial cells towards adipocytes in the presence of myoepithelium

**DOI:** 10.5713/ajas.18.0806

**Published:** 2019-04-15

**Authors:** Subi Sugathan, Sung-Jin Lee, Supriya Shiwani, Naresh Kumar Singh

**Affiliations:** 1 Department of Animal Biotechnology, College of Animal Life Sciences, Kangwon National University, Chuncheon 24341, Korea; 2 Department of Veterinary Surgery and Radiology, Faculty of Veterinary and Animal Sciences, Institute of Agricultural Sciences, Banaras Hindu University,Varanasi-221005, Uttar Pradesh, India

**Keywords:** Transdifferentiation, Peroxisomal Proliferator-activated Receptor γ (PPARγ), Linolenic Acid, Luminal Epithelium, Adipogenesis, Myoepithelial

## Abstract

**Objective:**

Orchastric changes in the mammary glands are vital, especially during lactation. The secretary epithelial cells together with the supporting myoepithelial and stromal cells function cordially to secrete milk. Increase in the number of luminal epithelial cells and a decrease in adipocytes are visible during lactation, whereas the reverse happens in the involution. However, an early involution occurs if the epithelial cells transdifferentiate towards adipocytes during the lactation period. We aimed to inhibit the adipocyte transdifferentiation of luminal cells by restraining the peroxisomal proliferator-activated receptor γ (PPARγ) pathway.

**Methods:**

Linolenic acid (LA) and thiazolidinediones (TZDs) induced adipogenesis in mammary epithelial cells were conducted in monolayer, mixed culture as well as in transwell plate co-culture with mammary myoepithelial cells.

**Results:**

Co-culture with myoepithelial cells showed higher adipogenic gene expression in epithelial cells under LA+TZDs treatment. Increase in the expressions of PPARγ, CCAAT/enhancer-binding protein α and vimentin in both mRNA as well as protein levels were observed. Whereas, bisphenol A diglycidyl ether treatment blocked LA+TZDs induced adipogenesis, as it could not show a significant rise in adipose related markers. Although comparative results were found in both mixed culture and monolayer conditions, co-culture technic was found to work better than the others.

**Conclusion:**

Antagonizing PPARγ pathway in the presence of myoepithelial cells can significantly reduce the adipogenisis in epithelial cells, suggesting therapeutic inhibition of PPARγ can be considered to counter early involution or excessive adipogenesis in mammary epithelium in animals.

## INTRODUCTION

The mammary fat pad adipocytes are essential for normal mammary gland development and vital for the postnatal development of mammary epithelium through the signal mediated morphogenesis of ducts [1–3]. Moreover, the function of adipocytes remain active in mammary gland throughout the lactation, however a visible adipocyte depletion is evident in lactation [4], which is further refilledduring involution [5]. On the other hand, the multiple layers cuboidal mammary epithelium will be composed to form the bulbous terminal end buds within the fat pad upon the pubertal hormonal responses [6]. Further proliferation and expansion of the epithelium and depletion of adipocytes will be happening due to reproductive hormonal stimulation during the lactation. However, when the nursing stops and suckling reduces, remodeling of mammary gland takes place through involution. In involution, some of the secretory epithelial will undergo apoptosis and the others will be redifferentiatedtowards adipocytes [7,8].

As the mammary gland is derived from both endodermal and mesodermal origins [9], the epithelial proliferation and development are crucially depend on mesenchymal interactions [10]. The fat pad in mammary gland provides vital signals that trigger the ductal morphogenesis and mammary development and its absence in rodents prevented the normal mammogenesis [2]. Further, unlike the other fat tissues, mammary fat pad expresses certain hormone receptors [11], which are related to the mammary development and thus the thyroxine induced treatment intensified the hormonal adipoblast-epithelial communication that is obligatory in each stage of developments in the gland [12].

Pubertal animals consist of adipose fat pad as a major com partment of mammary gland, whereas the mass of these fat pad tissues will be reduced by apoptosis and epithelial transdifferentiation in response to the hormonal regulations during pregnancy and lactation [13]. The lactating gland comprises of secretory epithelial tissue as dominant compartment, with a smaller fat pad. However, the fat pads are observed to be refilled through preadipocyte differentiation and adipogenesis of luminal cells during involution [4]. Therefore, to resist the luminal adipogenesis and thereby avoiding the precocious involution, we hypothesized to inhibit the major adipogenic specific peroxisomal proliferator-activated receptor γ (PPARγ) pathway with the help of an antagonist namely bisphenol A diglycidyl ether (BADGE).

## MATERIALS AND METHODS

### Chemicals, media, cells, and primers

Dulbecco’s modified Eagle’s medium (DMEM, high glucose), penicillin and streptomycin (PS) were purchased from Welgene (Dalseogu, Daegu, Korea), whereas fetal bovine serum (FBS) and horse serum are respectively from hyclone laboratories Inc (Logan, UT, USA) from Gibco Technologies Inc (Grand Island, NY, USA). Retinoic acid, linolenic acid (LA), dexamethasone, insulin, biotin, ascorbic acid, pantothenic acid, ascorbic acid, paraformaldehyde, hematoxyline, eosin, Triton X-100, 4′,6-diamidino-2-phenylindole dihydrochloride (DAPI) and fluoromount aqueous mounting media were obtained from Sigma Aldrich (St. Louis, MO, USA). Further, primary and secondary antibodies of both Donkey anti-goat immunoglobulin labelled with fluorescein isothiocyanate (IgG-FITC) and donkey anti-goat IgG-biotinylated were purchased from Santacruz biotechnology (Dallas, TX, USA) and primers from Macrogen.

### Mammary myoepithelial cell isolation

The myoepithelial cells from bovine mammary gland were isolated enzymatically according to the standard protocol. Briefly, the dissected glandular tissues from mammary gland were washed with phosphate buffered saline (PBS) several times before removing the fat tissues. After the removal of fat as much as possible, the tissues were then minced into fine pieces and treated with collagenase type II (300 U/mL) and hyaluronidase (100 U/mL) at 37°C for 3 hrs. The digested cells were strained with 40 μm cell strainer and washed with normal culture media and cultured in DMEM comprising 20% FBS and 1.1% PS. So isolated myoepithelial cells were proliferated and characterized using flow cyometric analysis with the help of BD FACSCalibur (BD-Biosciences, San Jose, CA, USA).

### Cell culture

#### Co culture

Epithelial cells, which were stored in LN_2_, were seeded and proliferated (in DMEM containing 10% FBS and 1.1% PS) till obtained a sufficient number of cells to perform the experiments. Total of 1×10^5^ epithelial cells were seeded into the bottom layer, whereas the myoepithelial cells were seeded proportionally in the top mesh of the Co culture plate. When the epithelial cells reached near confluency, the differentiation treatments were started through the initial induction (DMEM containing 2% horse serum, 1.1% PS, 50 μM ascorbic acid, 33 μM biotin, 10 μM acetic acid, 17 μM pantothenic acid, 0.5 μM 3-isobutyl-1-methylxanthine (IBMX), 1 μM dexamethasone, 10 μM insulin) for 24 hrs. Further, the treatment with 100 mM LA+10 μM thiazolidinedione (TZD) and 100 mM LA+10 μM TZD+100 μM BADGE were carried out for 48 hours.

*Mixed culture*: equal number of both myoepithelial and epithelial cells were mixed together and seeded into the 100 mm plastic cell culture plates. On nearby confluency, the cells were treated with initial induction media for 24 hours, and followed by the treatments with 100 mM LA+10 μM TZD and 100 mM LA+10 μM TZD+100 μM BADGE for 8 days, with media change of every 48 hours.

#### Monolayer

Total of 1×10^6^ epithelial cells were seeded into 100 mm dishes and the preconfluent dishes were treated with initial induction media followed by the 100 mM LA+10 μM TZD and 100 mM LA+10 μM TZD+100 μM BADGE. The treatment carried out till day 6 with media change in every 48 hours.

### Flow cytometric analysis

From the collected myoepithelial cells, small amount cells were fixed in 4% paraformaldehyde and permeabilized using triton X-100 (Sigma, Dorset, UK). These cells were stained with goat polyclonal primary antibodies, followed by the FITC conjugated donkey anti goat secondary antibodies. The flow cytometry was performed using FACS caliber and the data were analyzed using (Epics XL analyzer; Coulter Corporation, Miami, FL, USA).

### Immunostaining

The cell samples after the treatments were fixed in 4% paraformaldehyde before permeabilizing with Triton X-100. The permeabilized cells were incubated with skim milk blocker for blocking the unspecific proteins, followed by the staining with goat polyclonal primary antibodies (against mucin 1 [MUC1], keratin 18 [K18], K19, PPARγ, CCAAT/enhancer-binding protein α [C/EBPα] and vimentin proteins) and FITC labeled donkey anti goat secondary antibody. So stained cells were mounted in labeled glass slides with the help of flouromount gel. The prepared slides were visualized under the confocal laser microscope (Olympus FluoView FV1000, Tokyo, Japan) with the help of the Floueview confocal image analyzing software.

### Western blotting

The total protein from the collected cell samples was isolated using the proprep protein isolation kit (iNtRON Biotechnology, Seongnam, Korea). The harvested samples were washed with ice cold PBS before lysing with Proprep buffer. The lysed cell suspension was centrifuged to collect the total protein containing supernatant. These protein samples were mixed with the sodium dodecyl sulfate (SDS) reducing gel loading dye and heated in 95°C for 5 mins and placed in ice till loading for the SDS-polycrylamide gel electrophoresis (SDS-PAGE). Further the separated proteins in the gel were transferred into nitrocellulose membrane under electro-blotting arrangement. The free plots in the membrane were then blocked with a blocker solution (5% nonfat skim milk in PBS) to minimize the background. Further, the primary antibody incubation was carried out at 4°C overnight, followed by the biotinylated secondary antibody staining for 1 hour at room temperature. The biotin in the secondary antibody was then tagged with the streptavidin, which is conjugated with horse radish peroxidase enzyme. Finally, Opti-CN kit based detection was carried out and the visible bands were documented using an optical scanner. The protein bands were then analyzed using ImageJ software and the data were normalized to the internal loading control actin.

### Cytochemical staining

#### Ayoub shklar

The staining sample cells were fixed in 4% paraformaldehyde prior to staining. The fixed cells were treated with acid fuchsin stain for 5 mins, followed by the aniline blue and orange G (equal in volume) mixture for 40 mins. Further the stained cells were washed with 95% ethanol and air dried prior to take pictures.

#### Oil red O

The paraformaldehyde fixed cells were treated with 6:4 working solution of oil red O (ORO) for 10 mins. Further, washed thoroughly with water and taken the images under inverted light microscope. Finally, the stain was eluted out using 100% isopropanol, for determining the ORO concentration with the help of spectrophotometer.

#### Hematoxylin and eosin staining

Cells after fixing with paraformaldehyde were washed with distilled water, followed by the hematoxyline staining for 10 mins. The excess stains were washed out by washing with tap water and further stained with eosin for 5 mins. Finally, the stained samples were washed and dried using ethanol prior to take pictures under inverted light microscope.

### Reverse transcriptase polymerase chain reaction

The total RNA from the harvested samples were extracted using trizol reagent (Invitrogen; Life Technologies Inc., Grand Island, NY, USA). cDNA synthesis from the collected total RNA performed using the reverse transcription kit (RiverTra Ace, Toyobo, Osaka, Japan), under the standard protocols of manufacturer. So synthesized cDNA samples were used for the polymerase chain reaction (PCR) amplification under the programmed conditions, using different primers (Table 1), in the thermocycler. The standard program used for PCR amplification is, initial denaturation at 95°C for 5 mins, followed by 35 cycles of 95°C for 30 s, 58°C to 63°C for 30 s and 72°C for 30 s, and a final elongation at 72°C for 8 mins before storing on 4°C. The amplified products were then run in ethidium bromide stained 1% agarose gel and visualized under UV transilluminator documentation system. The bands obtained from the gels were analyzed using ImageJ software, further, the data were normalized to the expression of internal loading control glyceraldehyde 3-phosphate dehydrogenase.

### Statistical analysis

The treatments in different experimental techniques at different replicates were analyzed by analysis of variance, using the statistical software package SAS (SAS Inst. Inc., Cary, NC, USA). The significant differences were determined (p<0.05, p<0.01, p<0.001) by the Duncan’s multiple range tests using SAS.

## RESULTS

**Figure 1:**
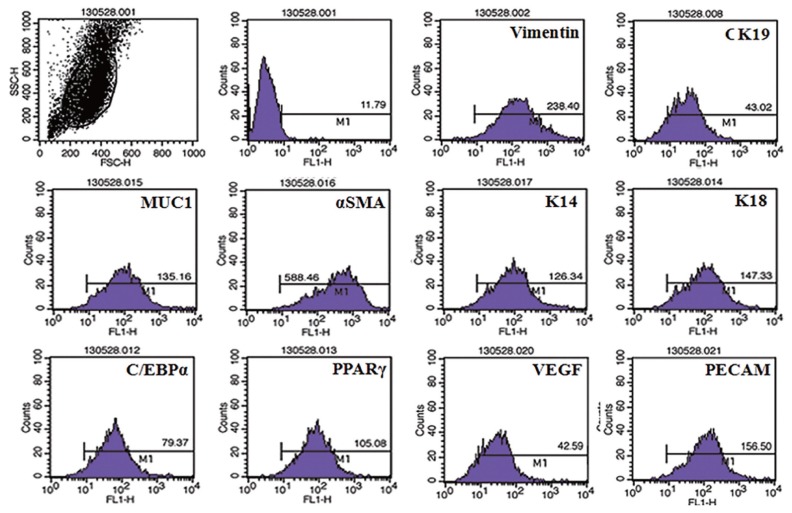
Flow cytometric characterization of mammary myoepithelial cells. Characterization of isolated bovine mammary myoepithelial cells using different markers. MUC1, mucin1; αSMA, α smotth muscle actin; K14, keratin 14; C/EBPα, CCAAT/enhancer-binding protein α; PPARγ, peroxisomal proliferator-activated receptor γ; VEGF, vascular endothelial growth factor; PECAM, platelet endothelial cell adhesion molecule.

The isolated myoepithelial cells showed predominant expressions of basal cell markers comparatively to the other mammary cell specific proteins. Significant expression of smooth muscke actin, K14, vimentin were visible in the isolated cells, whereas other luminal and stromal cell markers showed a little expression (Figure 1).

### Effect of treatments on luminal cells under the influence of basal cells in coculture conditions

**Figure 2:**
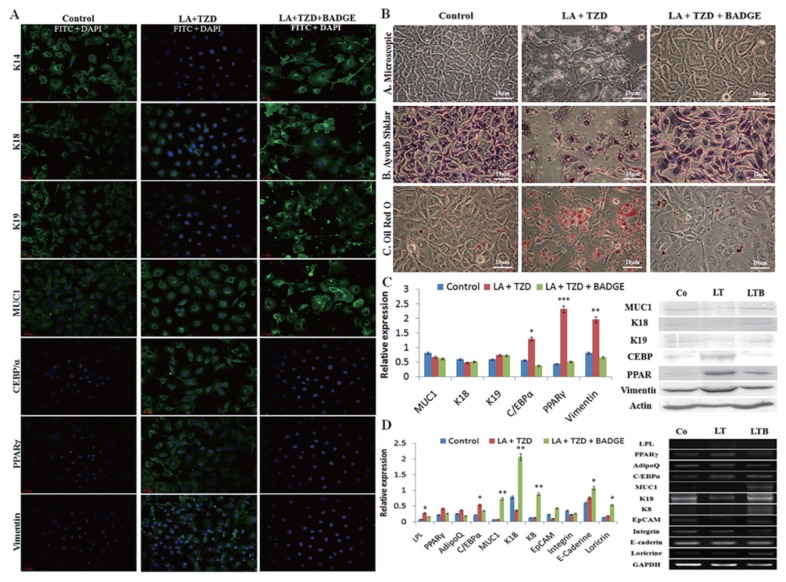
Adipocyte transdifferentiation of luminal cells under the influence of myoepithelial cells co culture. The adipogenic induction in luminal cells under the influence of myoepithelial cells revealed significant upregulation of fat specific markers. However, the inhibition of PPARγ signaling retarded adipose markers and enhanced/retained the luminal marker expression in immunostaining (A), cytochemical staining (B), immunoblotting (C), and Reverse transcriptase polymerase chain reaction (D) analysis. LA, linolenic acid; TZD, thiazolidinediones; BADGE, bisphenol A diglycidyl ether; Co, control; LT, LA+TZD; LTB, LA+TZD+BADGE; PPARγ, peroxisomal proliferator-activated receptor γ; LPL, lipoprotein lipase; C/EBPα, CCAAT/enhancer-binding protein α; MUC1, mucin1; K18, keratin 18; EpCAM, epithelial cell adhesion molecule. * p<0.05, ** p<0.01, *** p<0.001.

The epithelial cells while cocultured with the mammary myoepithelial cells adopted luminal fate in normal control conditions, where expressions of K18, K19, and MUC1 along with the basal marker K14 were visible significantly. However, there were no signs of trigger in adipogenic markers such as PPARγ, C/EBPα, vimentin detected on immunostaining (Figure 2A), immunoblotting (Figure 2C), and RT-PCR (Figure 2D). Further, the ayoub shklar staining showed positively stained epithelial cells and ORO unstained cells under control groups. In contrast, the LA+TZD treatment in luminal cells silenced the epithelial factors and enhanced the adipogenic transition. Under this treatment, intake of lipid droplets as well as the expressions of adipogenic markers was detected significantly enhanced.

However, the addition of BADGE in the induction media blocked the adipogenic impulse of luminal cells. The treatment of BADGE disrupted the PPARγ expression and, thereby, inhibited the other adipogenic factors such as C/EBPα and vimentin. Furthermore, the addition of BADGE in the adipogenic prompt media retained the luminal fate of epithelial cells, explained through the highly significant epithelial marker expressions in downstream analyses of genes and proteins as well as cytochemical stains (Figure 2B).

### Effect of treatments on luminal and basal cells under mixed culture conditions

**Figure 3:**
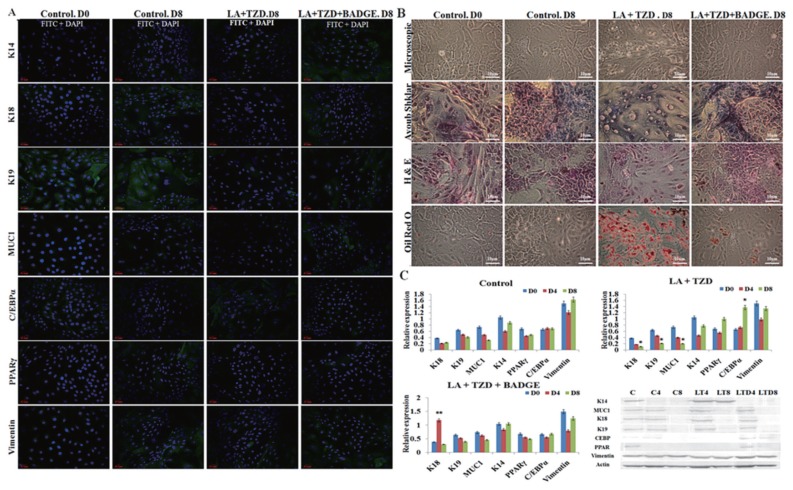
Adipogenic induction of luminal cells together with the myoepithelial cells in mixed culture. (A) and (C) The myoepithelial, epithelial and adipose specific marker protein expressions were analyzed through immunostaining and immunoblotting, where, downregulation of epithelial proteins in the control group (C0–C8) the, LA+TZD (LT4, LT8) and LA+TZD+BADGE (LTD4, LTD8) treated cells were monitored, thought the step up regulations in myoepithelial K14 in LTB and adipose proteins in LT treated cells were perceived. The documented bands within the membrane were analyzed through imageJ software and normalized to the internal loading control Actin (* p<0.05, ** p<0.01). (B) Morphological alterations as well as cytological changes in mixed culture cells under adipogenic inductions were evaluated using microscopic observations of ayoub shklar, H&E as well as oil red O stainings. Where smaller lipid intakes were shown in luminal cells under LA+TZD treatment, however, in the PPARγ blocked treatment, just like control, did not show any sign of adipose conversion even in the presence of LA+TZD. Further, ayoub shklar and H&E stained images showed fading luminal factors under PPARγ active groups and higher luminal stains under PPARγ inhibition. LA, linolenic acid; TZD, thiazolidinediones; BADGE, bisphenol A diglycidyl ether; H&E, hematoxylin and eosin; PPARγ, Peroxisomal proliferator-activated receptor γ; K18, keratin 18; MUC1, mucin1; C/EBPα, CCAAT/enhancer-binding protein α.

**Figure 4:**
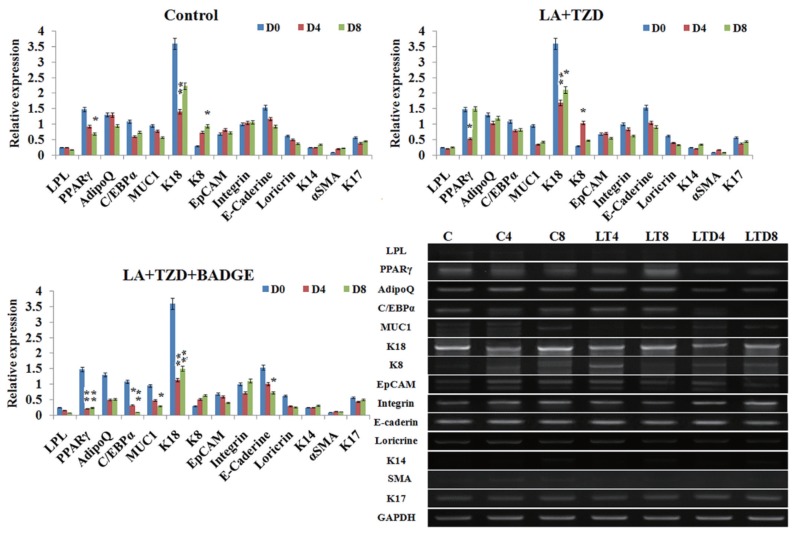
RT-PCR analysis for assessing variation in gene expressions upon adipocyte inductions in Epithelial and myoepithelial mixed culture. Adipocyte, epithelial and myoepithelial specific marker expressions under varied conditions of adipogenic inductions were detected using RT-PCR, where unchanged or negligible variations in epithelial marker gene expressions were observed in control (C0–C8) as well as PPARγ inhibited induction condition (LTB4, LTB8), in which, the adipocyte markers showed the gradual arrest of their expressions. However, in LA+TZD treatment (LT4, LT8), neither significant upregulation, nor downmodulations of adipocyte genes were observed. Although, slight downregulations of epithelial genes were detected. The normalization of each band was done with the expression of internal loading control GAPDH. * p<0.05, ** p<0.01. RT-PCR, reverse transcriptase polymerase chain reaction; PPARγ, peroxisomal proliferator-activated receptor γ; LA, linolenic acid; TZD, thiazolidinediones; GAPDH, glyceraldehyde 3-phosphate dehydrogenase; LPL, lipoprotein lipase; C/EBPα, CCAAT/enhancer-binding protein α; MUC1, mucin1; K18, keratin 18; K8, keratin 8; EpCAM, epithelial cell adhesion molecule; K14, keratin 14; αSMA, α smotth muscle actin; K17, keratin 17.

When the luminal and basal cells were cultured together in equal ratio, not much remarkable changes were noticed in control groups (Figure 3), where expressions of both luminal and basal markers showed balanced expressions (Figure 3A). However, when the cells were cultured under the presence of adipogenic inducers, disruption of luminal markers and insignificant change of basal K14 were noticed (Figure 3C). Further, the adipogenic specific markers like C/EBPα and PPARγ showed salient upregulation under the LA+TZD treatment (Figures 3A, 3C, 4) and the cytochemical staining of adipogenic induced cells observed tightly packed with the lipid droplets, though the cells likely myoepithelial in morphology rarely showed lipid inside. In addition, ayoub shklar and H&E staining showed negatively stained for epithelium after treatment (Figure 3B).

On the other hand, the disruption of PPARγ signaling inhi bited the adipogenic conversion in epithelial cells neighboring myoepithelium. The eight days of treatment could not trigger the adipogenic fate of luminal cells, whereas they maintained their own lineages under this particular treatment group. Changes in epithelial as well as basal markers were detected insignificant (Figures 3A, 3C, 4), moreover, cytochemical staining revealed the nondifferentiated cells in all ayoub shklar, H&E as well as ORO (Figure 3B).

### Effect of treatments on luminal epithelial cells in monolayer

**Figure 5:**
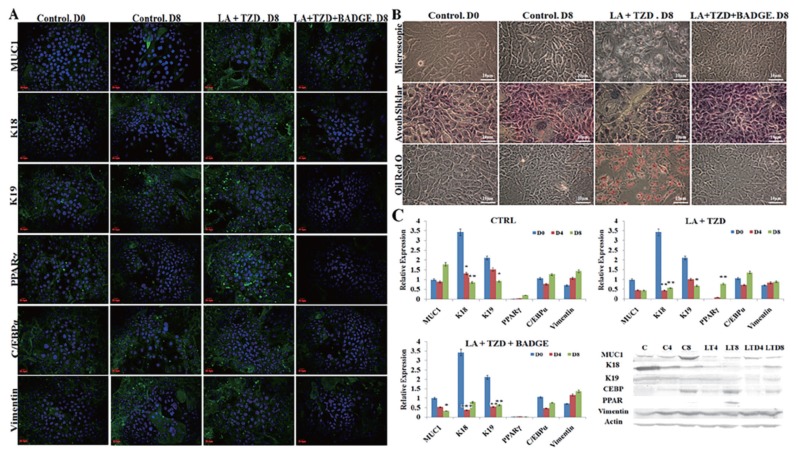
Adipogenesis of luminal cells in monlayer. (A) and (C) The epithelial and adipose specific marker protein expressions were analyzed through immunostaining and immunoblotting, where, adipogenic markers along with luminal markers were expressed under LA+TZD (LT4, LT8) treatment, similarly the control cells (C0–C8) also expressed both kinds of markers but significantly lower adipogenic markers. However, the LA+TZD+BADGE (LTD4, LTD8) treatment restricted the adipogenic induction as epithelial markers were still evident. The documented bands within the membrane were analyzed through imageJ software and normalized to the internal loading control Actin (* p<0.05, ** p<0.01). (B) The cells after the induction with adipogenic inducers monitored microscopically for morphological changes after ayoub shklar and oil red O staining. LA, linolenic acid; TZD, thiazolidinediones; BADGE, bisphenol A diglycidyl ether; MUC1, mucin1; K18, keratin 18; K19, keratin 19; PPARγ, peroxisomal proliferator-activated receptor γ; C/EBPα, CCAAT/enhancer-binding protein α.

**Figure 6:**
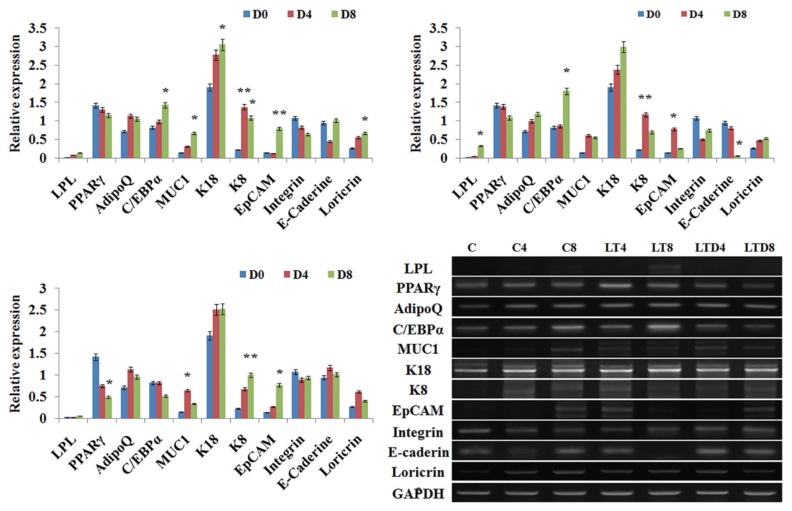
Reverse transcriptase polymerase chain reaction analysis of adipocyte and epithelial specific gene expressions in mammary epithelial cells under adipocyte differentiation conditions. Progressive upregulations of adipocyte genes in epithelial cells were observed under LA+TZD treatment (LT4, LT8), however, inhibition of PPARγ (LTD4, LTD8) blocked arrested the adipocyte gene expressions. Moreover, expressions of epithelial genes in control (C0–C8) cells were demodulated under adipocyte differentiation conditions. The normalization of each band was done with the expression of internal loading control glyceraldehyde 3-phosphate dehydrogenase (* p<0.05, ** p<0.01). LA, linolenic acid; TZD, thiazolidinediones; LPL, lipoprotein lipase; PPARγ, peroxisomal proliferator-activated receptor γ; C/EBPα, CCAAT/enhancer-binding protein α; MUC1, mucin1; K18, keratin 18; K8, keratin 8; EpCAM, epithelial cell adhesion molecule.

When the bovine mammary luminal cell cultures in monolayer, expressions of luminal markers, K18, K19, and MUC1 were monitored along with the slight expressions of adipocyte PPARγ and C/EBPα (Figure 5A). However, the treatment of LA+TZD on luminal cells enhanced the adipogenic marker expressions significantly (p<0.05) (Figure 5A, 5C, 6), thereby the lipid droplet formation within the cells were also intensified (confirmed by the elution index data for ORO staining). Moreover, the makeover of blue stains replacing the epithelial specific reddish dye explains the transdifferentiation of luminal cells from the epithelial lineage (Figure 5B). However, we did not attain the complete transition towards adipocyte lineage, where the expressions of luminal markers were also existed within the treated cells. Immuno-blotting data showed significant (p<0.05) step up regulation of PPARγ was observed in the treated cells, whereas the terminal adipogenic marker C/EBPα expression was not significant. In contrast, the RNA analysis revealed significance in C/EBPα regulation and silencing of epithelial markers (Figure 6).

Conversely, the treatment with BADGE inhibited both luminal and adipose specific markers in epithelial cells. The PPARγ antagonist disrupted the adipogenic fate even in the presence of adipogenic inducers, moreover, fading off of K18, K19, and MUC1 under BADGE was noticeable (Figure 5A, 5C, 6). Nevertheless, vimentin expression showed positively regulated under this particular treatment. Furthermore, the ayoub shklar staining gave competable similarities with the control epithelial cells. In addition, ORO staining and its elution index revealed the non adipogenic cells without any stained lipid droplets (Figure 5B) under the BADGE treatment.

## DISCUSSION

Plasticity of the mammary cell types plays an interesting part in mammary gland evolution, where the hormone regulated changes in luminal cells and stromal adipoblasts are remarkable during pregnancy, lactation and involution [13]. The major portion of mammary gland of a virgin animal covered by adipoblast, which will then make over to 90% of secretory epithelial cells during pregnancy and lactation by the transdifferentiation of adipoblast towards epithelium. However, the postlactational period refills the stromal fat pad through adipogenesis of luminal cells [13].

Our results provide the evidence that luminal epithelial cells under adipogenic inducers, such as LA, TZD’s [14,15] can transdifferentiate to the adipocytes, however the PPARγ inhibition restricted the adipogenesis conversion in luminal epithelial cells. The TZD+LA treatment in luminal cells induced the PPARγ pathway that initiates the process of adipogenesis through lipid accumulation, which will be backed up by the terminal differentiator C/EBPα [16,17]. Moreover, these two genes together enhances the expressions of hundreds of other adipose specific genes those regulates lipid metabolism, lipid storage and adipokine secretion [18].

The changes in epithelial cells were detected by analyzing the difference in certain reliable luminal specific molecular markers such as MUC1, K18, K19, and epithelial cell adhesion molecule (EpCAM). These molecular markers were abundantly expressed in luminal epithelial cells. EpCAM, which is localized in the basolateral membrane in normal epithelium, restricts their expression within epithelial cells. This molecular marker is being used to separate the completely differentiated epithelial cells from the cluster of cells [19]. Whereas, MUC1 is one of the mostly recognized molecular marker for epithelial cells, which is produced in the apical plasma membrane of lactating epithelial cells and involved in the cellular interactions as well as in signal transductions [20]. However, the K18 is one of the structural proteins which is being synthesized during the epithelial differentiation program, which is an epithelial specific molecular marker expressed in single layer epithelial tissues and characterize the differentiation compartment of mammary luminal epithelial cells [21]. The keratinocyte expression in the differentiating epithelia is initiated by the expression of primary cytokeratin proteins, for instance one of the simple keratins K18. Which is further supplemented by certain secondary keratinocytes, like [21] the luminal mammary epithelial cells are exclusively positive to the molecular markers such as EpCAM, MUC1, and K18, thus their consistant expressions are useful to identify the luminal epithelial cells *in vivo* [22], whereas their diminishing expressions under adipogenic induction denotes the change over of luminal fate.

We analyzed the changes in vimentin, which is a mesen chymal structural protein consisting of intermediate filament. The significant upregulation in vimentin under the adipogenic treatment shows the transition of luminal cells to mesenchymal adipocytes [23]. Similarly, the sustained E-caderin expression in control as well as BAGDE treated luminal cells explaines the unchanged luminal fate [23]. E-caderin is the transmembrane glycoprotein essential for the stable tissue architecture of epithelium. Whereas the expressions of E-caderin and vimentin are counter related, where one’s expression suppresses the other’s [24].

The conversion of preadipocyte to fully differentiated adi pocytes is possible without the influence of any exogenous ligands. However, the adipogenesis of other cell types is entirely depend on the ectopic expressions of PPARγ, which needs the exposure of certain exogenous ligands such as TZD’s, LA and so on [25]. Our initial induction media comprising of insulin, dimethyl sulfoxide and IBMX are the potent inducers if C/EBPβ and C/EBPδ, whereas these factors eventually results in the ectopic expressions of two vital terminal adipogenic differentiation regulators PPARγ and C/EBPα in non adipogenic cells [26]. Furthermore, the adipogenic differentiation at its molecular front is being operated in a feed-forward manner, where the initial induction of PPARγ influence the C/EBPα expression to produce the completely differentiated adipocytes [27].

The transition of epithelial cells towards adipocytes under the LA+TZD induction were further confirmed with the higher expressions of adipose specific lipoprotein lipase (LPL) as well as adipoQ. LPL is one of the early markers in adipogenic differentiation, which blocks the proliferation of cells and initiates differentiation [28]. This enzyme hydrolyzes the triglycerides and stores the free fatty acids within the tissue during the adipogenesis [29]. While, the secretary protein adipoQ, which is synthesized in adipocytes accelerates the adipogenic transition of luminal epithelial cells [30].

However, in mixed culture experiment, we did not observe any sign of adipogenesis in both luminal and basal cells except the initial lipid droplet formation. The mammary myoepithelial cells, which is also known as the natural tumor suppressor, block the mesenchymal transition of luminal cells [31]. Suggstively, the direct interaction of myoepithelial cells inhibited the luminal adipocyte conversion even under the potential adipogenic inducers. Although in monolayer experiments, the adipogenic induction resulted in lipid accumulation and significant upmodulations of PPARγ, indicating initial adipogenesis [32], the complete conversion of luminal cells were not achieved within the experimental period. Nevertheless, the significant expression of *C/EBPα* gene on the final stages showed that the prolonged induction could provide completed transdifferentiation of luminal cells into adipocytes. Whereas, the presence of BAGDE in induction media restricted the initiation of adipogenesis and blocked the adipogenic transition altogether. Contrastingly, the upmodulation of vimentin was detected in the luminal cells, suggesting the possible transdifferentiation of mature luminal cells towards the vimentin postitve myoepithelium [33].

On the other hand, the treatment with a synthetic ligand (BADGE) of PPARγ antagonized the adipogenic differentiation in luminal cells, by competing with the agonists such as TZD’s and LA. The complete blockage of PPARγ was not possible with the BADGE because of its low solubility and affinity compared to the agonists [34]. Furthermore, the expression of PPARγ at a minute level is essential for the successive lactation in ruminant as it helps to produce cystolic lipid droplets in luminal cells, which is essential for the milk fat formation and initiation of lactation [35]. Therefore, the use of synthetic antagonists like BADGE can block the excessive adipogenesis under lactation and a minimum of its expression to maintain efficient milk production.

In conclusion, the higher adipogenic transdifferentiation of luminal epithelium under adipogenic induction conditions can be inhibited through the treatment with synthetic PPARγ antagonistslike BADGE or its derivatives of similar chemical structure. Suggestively, the mammary gland/lactational defects such as invasion, precocious involution and so on can be regulated effectively by the inhibition of PPARγ pathway.

## Figures and Tables

**Table 1:** Primers used for reverse transcriptase polymerase chain reaction analysis

mRNA	Forward	Reverse
PPARγ	5′-ACGGGAAAGACGACAGACAAA-3′	5′-ACGGAGCGAAACTGACACC-3′
Adipo Q	5′-GATCCAGGTCTTGTTGGTCCTAA-3′	5′-GAGCGGTATACATAGGCACTTTCTC-3′
LPL	5′-TACCCTGCCTGAAGTTTCCAC-3′	5′-CCCAGTTTCAGCCAGACTTTC-3′
C/EBPα	5′-AGTCCGTGGACAAGAACAGC-3′	5′-GGTCATTGTCACTGGTCAGC-3′
K14	5′-TGATCAGCAGCGTGGAAGAG-3′	5′-TGATCAGCAGCGTGGAAGAG-3′
SMA	5′-GATCACCATCGGGAATGAACGC-3′	5′-CTTAGAAGCATTTGCGGTGGAC-3′
K17	5′-ACTTCCGCACCAAGTTTGAG-3′	5′-GCTTTCATCTCCTCCTCGTG-3′
E-cadherin	5′-CCAGGTGACCACACTTGATG-3′	5′-ATACACATTGTCCCGGGTGT-3′
Integrin b1	5′-TGTCGAGTGTGTGAGTGCAA-3′	5′-AGACTCCAAGGCAGGTCTGA-3′
Loricrin	5′-CACTCATCCTTCCTGGTGCT-3′	5′-GCCCCCGGAATACTTGATAC-3′
K18	5′-GCGAGAAGGAGACCATGCAA-3′	5′-AGAATTTGCAAAAATCTGAGCCCT-3′
MUC1	5′-CGCAGAACTACGCCAGTTTCC-3′	5′-AGAGCGGGTGGTCATGGA-3′
EpCAM	5′-CGGTCAGTGCCAGTGTACTT-3′	5′-TTGAAGAGCCCCTTGTCGTC-3′
K8	5′-ACCGGAACATCAACCGTCTC-3′	5′-TCCCGTAGGACGAAGTCAGT-3′
Vimentin	5′-CAAGTCCAAGTTTGCTGACC-3′	5′-TCATGTTCTGAATCTCATCCTG-3′
GAPDH	5′-GGCGTGAACCACGAGAAGTATAA-3′	5′-CCCTCCACGATGCCAAAGT-3′

PPARγ, peroxisomal proliferator-activated receptor γ; LPL, lipoprotein lipase; C/EBPα, CCAAT/enhancer-binding protein α; K14, keratin 14; SMA, smotth muscle actin; MUC1, mucin1; EpCAM, epithelial cell adhesion molecule; GAPDH, glyceraldehyde 3-phosphate dehydrogenase.
